# Mean arterial pressure-aneurysm neck ratio predicts the rupture risk of intracranial aneurysm by reflecting pressure at the dome

**DOI:** 10.3389/fnagi.2023.1082800

**Published:** 2023-02-01

**Authors:** Jie Shen, Kaiyuan Huang, Yu Zhu, Yuxiang Weng, Feng Xiao, Rajneesh Mungur, Fan Wu, Jianwei Pan, Renya Zhan

**Affiliations:** Department of Neurosurgery, The First Affiliated Hospital, College of Medicine, Zhejiang University, Hangzhou, Zhejiang, China

**Keywords:** unruptured intracranial aneurysms, mean arterial pressure-aneurysm neck ratio, phases, predict, prognosis

## Abstract

**Background and purpose:**

The unruptured intracranial aneurysm (UIA) has high disability and mortality rate after rupture, it is particularly important to assess the risk of UIA and to carry out individualized treatment. The objective of this research is to introduce a novel parameter to predict the rupture risk of UIA.

**Methods:**

A total of 649 patients with 964 intracranial aneurysms in our center were enrolled. A novel parameter named mean arterial pressure-aneurysmal neck ratio (MAPN) was defined. Ten baseline clinical features and twelve aneurysm morphological characteristics were extracted to generate the MAPN model. The discriminatory performance of the MAPN model was compared with the PHASES score and the UCAS score.

**Results:**

In hemodynamic analysis, MAPN was positively correlated with wall shear stress and aneurysm top pressure, with Pearson correlation coefficients of 0.887 and 0.791, respectively. The MAPN was larger in the ruptured group (36.62 ± 18.96 vs. 28.38 ± 14.58, *P* < 0.001). The area under the curve (AUC) of the MAPN was superior than the AUC of aspect ratio (AR) and the bottleneck factor (BN), they were 0.64 (*P* < 0.001; 95% CI, 0.588–0.692), 0.611 (*P* < 0.001; 95% CI, 0.559–0.663) and 0.607 (*P* < 0.001; 95% CI, 0.554–0.660), respectively. The MAPN model constructed by aneurysm size, aneurysm location, presence of secondary sacs and MAPN, demonstrated good discriminatory ability. The MAPN model exhibited superior performance compared with the UCAS score and the PHASES score (the AUC values were 0.799 [*P* < 0.001; 95% CI, 0.756–0.840], 0.763 [*P* < 0.001; 95% CI,0.719–0.807] and 0.741 [*P* < 0.001; 95% CI, 0.695–0.787], respectively; the sensitivities were 0.849, 0.758 and 0.753, respectively).

**Conclusions:**

Research demonstrates the potential of MAPN to augment the clinical decision-making process for assessing the rupture risk of UIAs.

## Introduction

Intracranial aneurysm is a gradual abnormal distension of the local vascular wall arising from congenital dysplasia or acquired injury in the intracranial arterial lumen (Wiebers et al., 2003; de Rooij et al., 2007). The overall morbidity of intracranial aneurysms in the global adult population (with an average age of 50 years) was ~3.2% (Vlak et al., 2011). A retrospective study of 122916 UIAs from the United States found that the number of newly detected UIA patients increased by 1987 every year (Luther et al., 2020). The results of the international study of unruptured intracranial aneurysms (ISUIA) phase I study showed that the annual rupture rate of UIAs was 0.95% (Akio et al., 2012). The European Stroke Association's guidelines for intracranial aneurysms and SAH estimated that the annual risk of rupture of UIA in the general adult population was at least 1% (Steiner et al., 2013). Although the annual rupture rate of UIAs varies among the current studies, the overall rupture rate is between 1 and 2%, with an average of 2–16/100,000 people per year (Akio et al., 2012; Steiner et al., 2013). Ruptured intracranial aneurysms lead to subarachnoid hemorrhage (SAH), with a mortality rate of up to 45% and irreversible neurological dysfunction in more than half of the survivors (Wiebers et al., 2003). Therefore, it is particularly important to predict the rupture risk of intracranial aneurysms after detection to guide the treatment.

In recent years, rupture risk factors of UIAs have been extensively studied, including patient specific clinical characteristics, such as smoking, gender, age, hypertension and genetic components (Greving et al., 2014; Tominari et al., 2015; Backes et al., 2017; Bourcier et al., 2018; Juvela, 2019), as well as morphological characteristics of UIAs, such as the maximum size, location of aneurysms, secondary sacs of aneurysms, AR, BN and size ratio (SR) (Dhar et al., 2008; Ryu et al., 2011; Amenta et al., 2012). However, since the correlation between these risk factors and the status of UIAs is complex and multifactorial, no consensus has been reached, and stability assessment remains difficult. Compared with morphological factors and clinical characteristics, the pressure and hemodynamic parameters at the dome of the IAs can better reflect the stress state of the intracranial aneurysms and play a crucial role in the development and rupture of the intracranial aneurysms (Shojima et al., 2004; Miura et al., 2013; Hou et al., 2020). In this study, we combined the two parameters of mean arterial pressure and aneurysm neck width to define a new parameter, the ratio of mean arterial pressure to neck width, and deduced its relationship with aneurysm static pressure based on Bernoulli's law and hemodynamic analysis. Its ability to predict the rupture risk of UIAs was evaluated in clinical data. The discriminant ability of the model constructed by introducing new parameters was compared with UCAS score and PHASES score. It is further confirmed that MAPN makes a significant contribution to the judgment of IA stability.

## Methods

### Patient population

**Figure 1:**
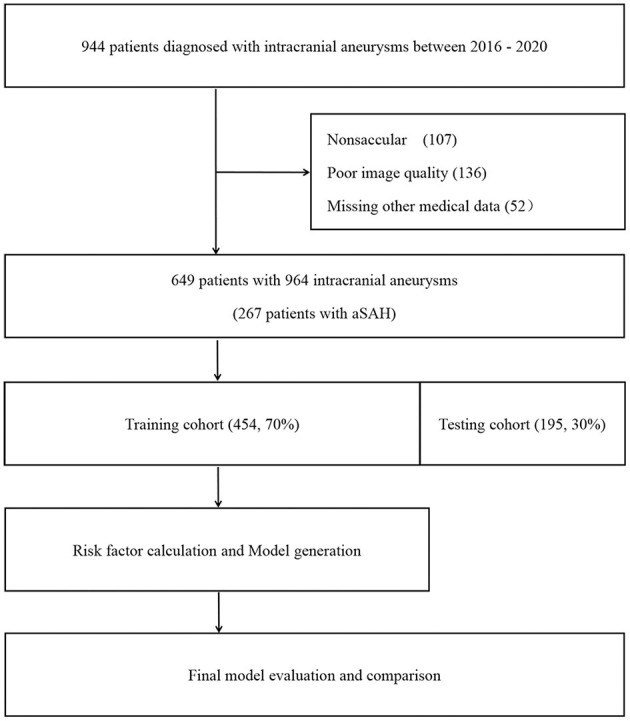
Study flow diagram. aSAH, aneurysmal subarachnoid hemorrhage.

This research cohort included 649 patients with intracranial aneurysms diagnosed and treated at the Department of Neurosurgery of our center from January 2016 to December 2020. This study was approved by the Ethics Review Committee of the First Affiliated Hospital of Zhejiang University School of Medicine. The whole cohort was randomly divided into a training set (*n* = 454) and a testing set (*n* = 195) by R language at a ratio of 7:3 prior to analysis. We retrospectively reviewed the medical records and imaging data in our database. The inclusion criteria were as follows: (1) saccular aneurysms confirmed on digital subtraction angiography (DSA), three-dimensional computed tomography angiography (CTA) or magnetic resonance angiography (MRA); (2) family members of patients who signed informed consent to cooperate with the clinical treatment procedures; (3) patients without surgical treatment in referral centers; and (4) age >18 years old. The exclusion criteria were as follows: (1) traumatic, infectious, fusiform, or dissecting aneurysms; (2) patients with moyamoya disease or vascular malformation; (3) absence of patients' important medical information; and (4) poor image quality or absence of important image records. The patient selection process is shown in Figure 1.

### Clinical and morphological features

The patient's clinical variables were collected as follows: (1) The demographic characters included: gender and age. (2) Medical history included: hyperlipidemia, hypertension, diabetes mellitus, history of cerebrovascular disease, alcohol consumption, smoking, antiplatelet (chronic low-dose) and family history of SAH. (3) Aneurysm morphology included: maximum size of aneurysm (the largest distance within aneurysm sac) (Dhar et al., 2008), neck width of aneurysm, height of aneurysm (the maximum perpendicular distance of the dome from the neck plane) (Dhar et al., 2008), width of aneurysm (the maximum distance of dome perpendicular to perpendicular height) (Dhar et al., 2008), multiple aneurysms, location of aneurysm, bifurcation position and presence of secondary sacs. The locations of aneurysms in our research were divided into three categories according to the hazard ratio of aneurysm location in the UCAS study (Akio et al., 2012): (a) ICA, VA; (b) ACA, MCA, PCA,BA; (c) ACom, IC-PCom. (4) The morphological parameters included: aspect ratio (AR), bottleneck factor (BN) and mean arterial pressure-aneurysm neck ratio (MAPN). To obtain accurate and objective measurement results, the morphological characteristics of IAs in this study were measured from reconstructed 3D CTA or DSA. Without considering the patient information, two neurosurgeons independently evaluated the morphological characteristics before grouping; Any inconsistencies were resolved by the third chief neurosurgeon (with more than 10 years of experience). AR, BN, and MAPN were calculated by the ratio of aneurysm height to neck width, aneurysm width to neck width, and mean arterial pressure to neck width, respectively (Ryu et al., 2011). Mean arterial pressure was calculated from 1/3 systolic blood pressure (SBP) plus 2/3 diastolic blood pressure (DBP). The blood pressure value in this study was obtained by consulting the outpatient records or health check-up center records. The dome of aneurysm is defined as the farthest point to the aneurysm neck.

### CFD simulation of patient specific geometries

We exported the DICOMs files sliced from 3-D DSA (Philips) to the Mimics Medical software (v21, Materialize) for aneurysm model reconstruction. The procedures for the extraction of surface geometry data from Mimics, and subsequent volume grids were generated using Geomagics. According to the previous studies, boundary conditions of inlets significantly affects only the region close to the inlet, and differences in flow solution are minimal beyond two diameters distal to the inlet where the flow is fully developed. Since patient specific flow information were not available for the present study, steady flow stimulations were applied using CFX software program (version 15.0) (Ansys, Inc, Lebanon, NH) in our patient-specific models. In boundary condition settings, mean velocity of 0.27 m/s was implied for proximal ICA inlets, a static pressure of 10,000 Pa were implied for the distal outlets. The artery walls were set as rigid. Blood flow was modeled as an incompressible, constant, and Newtonian fluid, that described by the Navier-Stokes equations in 3D. The blood density was 1,060 kg/m3, and the blood dynamic viscosity was 0.004 kg/m-s (Perktold and Peter, 1989; Xiang et al., 2011). In this study, mesh was calculated in ICEM–CFD, Tetrahedral and wall prism elements consisting of ~2,000,000 meshes with the element size of 0.1 mm were created for each aneurysm model using the ICEM–CFD meshing tool.

### PHASES score system

The PHASES score (ranging from 0 to 22) was developed in a study based on Finnish, Japanese and Dutch populations to predict the risk of aneurysm rupture and is now commonly used in patient management (Greving et al., 2014). This score was composed of 6 parameters, including population, hypertension, age, aneurysm size, early SAH and aneurysm locations.

### UCAS score system

This score system derivate from the Unruptured Cerebral Aneurysm Study (UCAS) in Japan, including patients with unruptured cerebral aneurysmsenrolled between 2000 and 2004 at neurosurgical departments at tertiary care hospitals in Japan (Tominari et al., 2015). Different from the PHASES score, this score increases the morphological factors of aneurysms, risk factors include age, sex, hypertension, aneurysm size, aneurysm location and daughter sac of aneurysm.

### MAPN model development and validation

A nomogram-risk model was generated based on the output variables processed by the binary logistic regression, which were initially selected and evaluated by univariate logistic regression. The discriminant performance of the model was measured by receiver operating characteristic (ROC) curves and the corresponding area under the curve (AUC). The applicability of the model was assessed by the Hosmer–Lemeshow test (*P* > 0.05 is considered a good fit), and the calibration diagram graphically illustrates the consistency between the predicted probability of rupture and the observed probability of rupture. In addition, the clinical net benefit of the model (a value of 0 indicates no benefit and a higher value indicates more benefit) was quantified, so as to demonstrate clinical effectiveness through decision curve analysis (DCA). R Studio software (version 4.2.0, Boston, MA, USA) for model generation, validation, and diagramming.

### Statistical analysis

Data were analyzed with SPSS, version 23.0 (IBM, Armonk, New York). Continuous variables were reported as the mean ± standard deviation and compared between ruptured and unruptured outcomes using an unpaired *T*-test. Categorical variables were reported as a proportion and percentile and were analyzed using *X*^2^ or Fisher's exact test, as appropriate. Univariate and multivariate logistic regression analyses were performed using ruptured as the outcome variable in the training cohort. Variables with a *P*-value ≤0.1 in univariate analysis were entered into binary logistic regression with stepwise backward selection.

## Results

### Basic patient information

The detailed processes of the selection and exclusion of the patients in the training cohort and testing cohort are shown in Figure 1. Finally, 454 patients were included in the training set, while 195 patients were included in the testing set.

In the training set, 192 (42.3%) patients were male, and 262 (57.7%) were female. The patients' ages ranged from 20 to 85, and ~12.3% of the patients were over 70 years old. There was no significant difference in age or gender between the rupture group and the unrupture group. The medical histories in the training set are summarized in Table 1. Diabetes mellitus, cerebrovascular disease, alcohol consumption, smoking and chronic history of low-dose antiplatelet were more common among those with ruptured intracranial aneurysms (*P* < 0.05).

**Table 1:** Baseline characteristics and aneurysm morphology of study cohorts.

**Variable**	**Training cohort (454)**		***P*-value**	**Testing cohort (195)**		***P*-value**
	**Unruptured**	**Ruptured**		**Unruptured**	**Ruptured**	
	**%/Mean** ±**SD**	**%/Mean** ±**SD**		**%/Mean** ±**SD**	**%/Mean** ±**SD**	
No. of patients	268(59%)	186(40.9%)		114(58.5%)	81(41.5%)	
**Demographic characters**						
**Age(years)**			0.142			0.456
<70	240(89.6%)	158(84.9%)		94(82.5%)	70(86.4%)	
≥70	28(10.4%)	28(15.1%)		20(17.5%)	11(13.6%)	
**Gender**			0.221			0.271
Male	107(39.9%)	85(45.7%)		50(43.9%)	42(51.9%)	
Female	161(60.1%)	101(54.3%)		64(56.1%)	39(48.1%)	
**Medical history**						
**Hyperlipidemia**	66(24.7%)	39(21.1%)	0.368	28(24.6%)	21(25.9%)	0.829
**Hypertension**	121(45.1%)	82(44.1%)	0.823	63(55.3%)	42(51.9%)	0.638
**Diabetes mellitus**	27(10.1%)	8(4.3%)	**0.023**	11(9.6%)	6(7.4%)	0.584
**Cerebrovascular disease**	45(16.8%)	14(7.5%)	**0.004**	16(14%)	2(2.5%)	**0.006**
**Alcohol consumption**	35(13.1%)	40(21.5%)	**0.017**	16(14%)	21(25.9%)	**0.037**
**Smoking**	41(15.3%)	45(24.2%)	**0.017**	23(20.3%)	23(28.4%)	0.183
**Antiplatelet (chronic low-dose)**	38(14.2%)	9(4.8%)	**0.001**	14(12.3%)	1(1.2%)	**0.004**
**Family history of SAH**	3(1.1%)	1(0.5%)	0.514	0	3()	**0.038**
**Aneurysm morphology**						
**Maximum size (mm)**	6.15 ± 3.77	6.77 ± 3.52	0.075	6.15 ± 4.16	6.79 ± 3.37	0.253
**Neck width (mm)**	4.33 ± 2.61	3.52 ± 2.41	**0.001**	4.03 ± 2.49	3.35 ± 1.33	**0.026**
**Height (mm)**	4.87 ± 3.22	4.68 ± 2.87	0.533	4.75 ± 3.64	4.55 ± 2.45	0.659
**Width (mm)**	5.19 ± 3.66	4.77 ± 3.09	0.209	5.23 ± 3.80	4.71 ± 2.89	0.307
**Aspect ratio (AR)**	1.22 ± 0.64	1.49 ± 0.84	**<0.001**	1.30 ± 0.83	1.44 ± 0.68	0.216
**Bottleneck factor (BN)**	1.24 ± 0.58	1.46 ± 0.71	**<0.001**	1.36 ± 0.67	1.54 ± 1.42	0.248
**Mean arterial pressure-aneurysm neck ratio (MAPN)**	28.38 ± 14.58	36.62 ± 18.96	**<0.001**	30.34 ± 15.72	36.92 ± 20.41	**0.012**
**Size of aneurysm**			**0.028**			0.104
<7mm	193(72%)	114(61.3%)		86(75.4%)	49(60.5%)	
7 mm ≤size <10 mm	43(16%)	39(21%)		17(14.9%)	17(21%)	
10 mm ≤size <20 mm	29(10.8%)	30(16.1%)		9(7.9%)	14(17.3%)	
≥20 mm	3(1.1%)	3(1.6%)		2(1.8%)	1(1.2%)	
**Location of Aneurysm**			**<0.001**			**<0.001**
ICA, VA	131(48.9%)	25(13.4%)		54(47.4%)	10(12.3%)	
ACA, MCA, PCA,BA	54(20.1%)	49(26.3%)		15(13.2%)	30(37%)	
ACom, IC-PCom	83(31%)	112(60.2%)		45(39.5%)	41(50.6%)	
**Bifurcation**	49(18.3%)	58(31.2%)	**0.001**	19(16.7%)	23(28.4%)	**0.05**
**Presence of secondary sacs**	57(21.3%)	85(45.7%)	**<0.001**	33(28.9%)	40(49.4%)	**0.004**

SAH, Subarachnoid hemorrhage; AR, Aspect ratio; BN, Bottleneck factor; MAPN, ratio of mean arterial pressure to aneurysm neck; ICA, Internal carotid artery; VA, Vertebral artery; ACA, Anterior cerebral artery; MCA, Middle cerebral artery; BA, Basilar cerebellar artery; ACom, Anterior communicating artery; IC-PCom, Internal carotid-posterior communicating artery.

In the testing set, 92 (47.2%) patients were male and 103 (52.3%) were female. Among these patients, the age range was 22–83 years, and ~15.9% of the patients were over 70 years old. There was no significant difference in age or gender between the rupture group and the unrupture group. The medical histories in the testing set are summarized in Table 1, which shows that diabetes mellitus, cerebrovascular disease, chronic history of low-dose antiplatelet therapy and family history of SAH were more common among those with ruptured intracranial aneurysms (*P* < 0.05).

### Aneurysm morphological characteristics

The morphological features of the UIAs and ruptured IAs are shown in Table 1. Aneurysms were classified by maximum size: 442 were small (<7 mm), 116 were moderate (7–10 mm), 82 were large (10–20 mm) and 9 were giant (≥20 mm). Aneurysm size was significantly different between the ruptured and unruptured groups in the training set, but no significant differences were observed in the testing set. AR and BN also showed statistically significant differences between the ruptured group and the unruptured group in the training set, but such significant differences did not exist in the testing set, which may be caused by the small number of testing sets. There were statistically significant differences (*P* < 0.05) in neck width, MAPN, location of aneurysm, bifurcated aneurysm, and presence of secondary sacs between the ruptured and unruptured groups in both the training set and the testing set.

### Relationship between MAPN and the pressure at the aneurysmal dome

**Figure 2:**
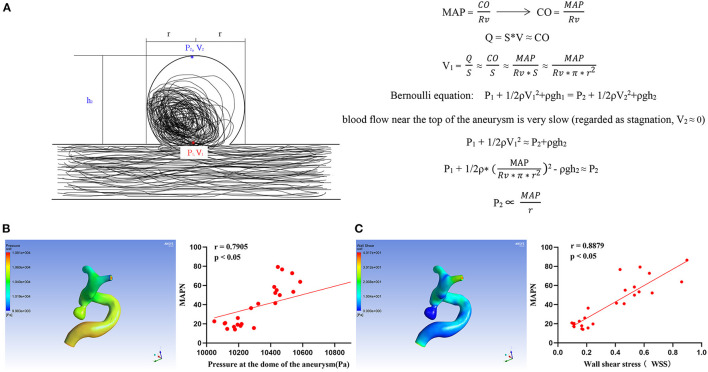
**(A)** Formula derivation of the relationship between MAPN and pressure at aneurysm dome MAP, mean arterial pressure; CO, cardiac output; Rv, peripheral resistance; Q, liquid flow; S, pipe diameter area; V, liquid flow rate; P_1_ and P_2_ are the static pressure, ρ, density; h, vertical height; g, acceleration of gravity. **(B, C)** Relationship between MAPN and wall shear stress and aneurysm pressure at the dome, respectively.

In Figure 2, the linear proportion relationship between MAPN and static pressure of intra-aneurysms was deduced by the Bernoulli equation. It shows that MAPN, a new parameter, can reflect the changes of blood pressure in the aneurysm cavity. The specific formula derivation process can be sawed in the Supplementary material. In the hemodynamic analysis of IAs, it was also found that MAPN was positively correlated with wall shear stress (WSS) and the pressure at the dome of aneurysm.

### Diagnostic ability of MAPN in aneurysm rupture risk

**Figure 3:**
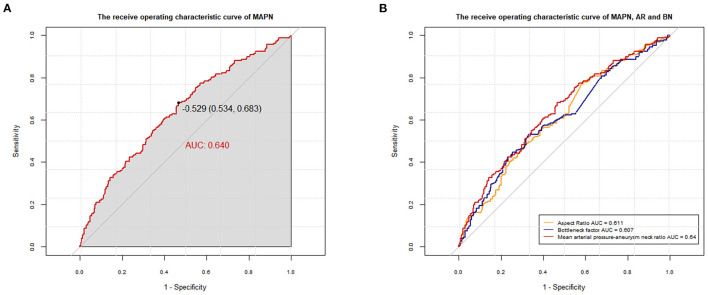
**(A)** The AUC of the MAPN is 0.640 (*p* < 0.001; 95% CI, 0.588–0.691) in the training set. **(B)** Comparison of the diagnostic capabilities between MAPN, AR and BN, and their AUC values were 0.64, 0.611 (*p* < 0.001; 95% CI, 0.559–0.664) and 0.607 (*p* < 0.001; 95% CI, 0.554–0.660), respectively.

In the univariate analysis, the mean MAPN value of the rupture group was higher in both sets (*P* < 0.05). In Figure 3A, the AUC of the MAPN is 0.64, demonstrating that this new parameter has a certain diagnostic ability for aneurysm rupture. Its cutoff point value is −0.529 (0.534, 0.683). In Figure 3B, we compared the diagnostic ability of MAPN with AR and BN, and their AUC values were 0.64, 0.611 and 0.607, respectively, suggesting that MAPN is superior to AR and BN in the diagnosis of aneurysm rupture.

### Results of the binary logistic regression and the MAPN model

**Figure 4:**
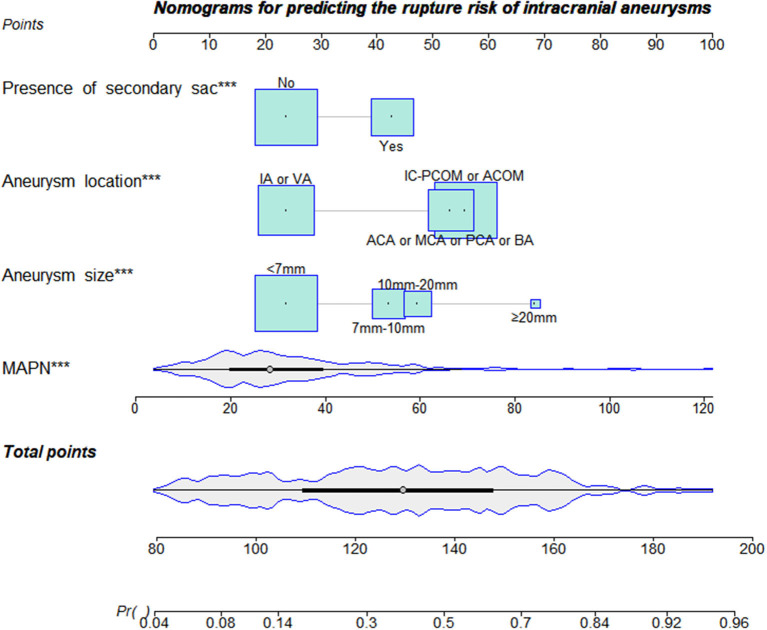
The nomogram was plotted based on 4 independent factors. The size of the bars indicates the number for each category, and the density plot represents the population distribution of each continuous variable. The total points, derived from the sum points of the four individual indexes, determined the risk of aneurysm rupture.

Thirteen variables (*P* < 0.05 in univariate analysis) were entered into multivariate logistic regression. Table 2 shows the diabetes mellitus (OR, 0.345; *p* = 0.025), cerebrovascular disease (OR, 0.290; *p* = 0.001), presence of secondary sacs (OR, 2.806; *p* < 0.001), BPN (OR, 1.057; *p* < 0.001), size of aneurysm and location of aneurysm are independent influencing factors for rupture of cerebral aneurysms (Table 2). The Hosmer–Lemeshow test reflects a satisfied degree of consistency between the predicted risk of the model and the actual risk (*P* = 0.695, Table 2). Significant differences were found in the subgroup analysis of diabetes, cerebrovascular disease and drug use, suggesting that there may be an interaction between these parameters (Supplementary material). Thus, diabetes and cerebrovascular disease were excluded from the construction of the final MAPN prediction model. As shown in Figure 4, the final MAPN prediction model included the presence of secondary sacs, size of aneurysm, location of aneurysm and MAPN.

**Table 2:** Logistic regression table for risk factors associated with rupture of cerebral aneurysms.

**Variable included in model**	**OR**	**95%CI**	***P***
Diabetes mellitus	0.345	0.136–0.877	0.025
Cerebrovascular disease	0.290	0.136–0.617	0.001
Presence of secondary sacs	2.806	1.710–4.606	<0.001
**Size of aneurysm**			
**<7mm**	**Reference**		
7 mm ≤size <10mm	3.428	1.759–6.683	<0.001
10 mm ≤size <20mm	5.406	2.244–13.025	<0.001
≥ 20mm	23.042	3.040–174.625	0.002
**Location of aneurysm**			
**ICA, VA**	**Reference**		
ACA, MCA, BA	5.055	2.660–9.607	<0.001
ACom, IC-PCom	6.390	3.040–11.428	<0.001
MAPN	1.057	1.038–1.077	<0.001
**Hosmer and Lemeshow test**			
*X* ^2^			5.575
Degree of freedom			8
*P*			0.695

### Comparison of discriminatory models

**Figure 5:**
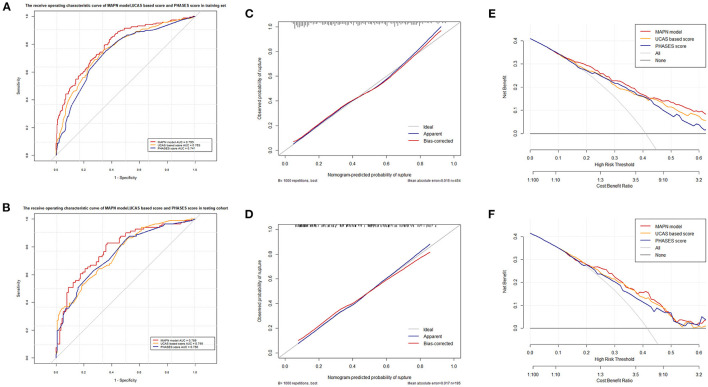
**(A, B)** shows the different AUC values of the three prediction models in the training and validation sets, respectively. **(C, D)** shows the different calibration curves of the three prediction models in the training and validation sets, respectively. **(E, F)** shows the different decision curve analyses of the three prediction models in the training and validation sets, respectively.

The ROC curve and AUC value of each model in the training set and the testing set are shown in Figure 5. In the training set, the MAPN model showed the best performance, with an AUC value of 0.799 (95% CI, 0.756–0.840), and the AUC values of the UCAS score and the PHASES score were 0.763(95% CI, 0.719–0.807), and 0.741 (95% CI, 0.695–0.787), respectively. In the testing set, the MAPN model also performed well with an AUC value of 0.786 (95% CI, 0.721–0.851), and the AUC values of the UCAS score and the PHASES score were 0.755(95% CI, 0.688–0.822) and 0.750 (95% CI, 0.681–0.819), respectively. The calibration plot showed good overall agreement between the predicted risk and observed events. Decision curve analysis suggested that given any selected risk threshold, the MAPN model achieved more benefit than both the UCAS score and PHASES score. In addition, Table 3 also suggests that the MAPN model has higher sensitivity and accuracy in the diagnosis of aneurysm rupture.

**Table 3:** Performance of each model in training cohort and testing cohort.

**Model**	**Sensitivity**	**Specificity**	**Accuracy**	**AUC (95%CI)**
**Training cohort**				
MAPN model	0.849	0.604	0.705	0.799 (0.756–0.840)
UCAS score	0.758	0.657	0.698	0.763 (0.719–0.807)
PHASES score	0.753	0.642	0.687	0.741 (0.695–0.787)
**Testing cohort**				
MAPN model	0.827	0.632	0.713	0.786 (0.721–0.851)
UCAS score	0.815	0.535	0.651	0.755 (0.688–0.822)
PHASES score	0.630	0.728	0.687	0.750 (0.681–0.819)

## Discussion

The degeneration of the vascular wall caused by abnormal hemodynamic changes is also an important reason for the formation of intracranial aneurysms. Current studies have shown that various hemodynamic parameters such as WSS, oscillatory shear index (OSI), blood flow impact, blood flow velocity and static pressure play an important role in the formation, enlargement, rupture and recurrence of intracranial aneurysms (Xiang et al., 2011; Kerl et al., 2014; Cebral et al., 2016; Xiao et al., 2018). In this research, the linear proportion relationship between MAPN and static pressure of intra-aneurysms was deduced by the Bernoulli equation, which provides a theoretical basis for MAPN to accurately predict the rupture risk of IAs. However, in reality, the fluid flow inside the aneurysm is often turbulent (Hiroshi et al., 2001; Steinman and Pereira, 2019), which is more complicated than the conditions we assumed. Therefore, this derivation also has certain limitations. In general, the application of MAPN could strengthen the clinical decision-making process for evaluating the risk of UIAS rupture.

When the MAPN parameter is used to predict intracranial aneurysm rupture, the MAP should be taken from the individualized daily average of patients to judge the long-term rupture risk. Previous studies used a history of hypertension as a risk factor in predicting the risk of aneurysm rupture (Brown and Broderick, 2014; Greving et al., 2014; Tominari et al., 2015), however, individualized blood pressure (BP) value is more valuable than hypertension history. Bakker et al. proposed the linear effect of BP on intracranial aneurysm liability. For BP traits, there is an 8–12% increase in the intracranial aneurysm risk per each mmHg increase in the DBP and a 3.7–6.0% increase in the intracranial aneurysm risk per each mmHg increase in SBP (Bakker et al., 2020). This finding suggests the important role of individual blood pressure values in the development and growth of intracranial aneurysms. Similarly, Hasan et al. proposed that acute hypertension, will increase intra-aneurysmal pressures and hence result in an increase in stress placed upon the aneurysmal walls, and the patients' individual blood pressure is often more valuable than a history of hypertension in predicting the rupture risk of IAs (Hasan et al., 2015). For the aneurysmal neck, it is indisputable that the ostium area plays a central but complex role in flow dynamics: narrower ostia promote jetting and flow instability but also allows for resistance to flow entering the sac, suppressing jet momentum and instability (Steinman and Pereira, 2019). The MAPN value will change dynamically with the patient's blood pressure management and when there is growth of intracranial aneurysm, which indirectly reflects the change in the static pressure of the intracranial aneurysm dome and can achieve the dynamic assessment of the rupture risk of IAs.

The prediction ability of the rupture of IAs is still acceptable according to the performance of MAPN in the training set; at least, its predictive ability is better than AR and BN. The neck width was incorporated into all three parameters. Part of the reason is that the neck region is a factor that restricts the blood flow into and out of aneurysms and has a certain relationship with the speed of blood flow into and out of aneurysms (Hiroshi et al., 2001; Saqr et al., 2020). In the actual clinical measurement process, the morphological parameters of aneurysms are affected by the thrombus around the aneurysms or the thrombus in the aneurysms (Hiroshi et al., 2001), which leads to the underestimation of the measured values. Therefore, the real difference of AR and BN between ruptured aneurysms and unruptured aneurysms may be larger, while the measurement error of the mean arterial pressure in MAPN is relatively smaller, which may be the reason why the prediction ability of MAPN is better than that of AR and BN.

Diabetes and cerebrovascular disease history were removed from the MAPN model because diabetes and cerebrovascular disease history became protective factors against aneurysm rupture. The same phenomenon has been seen in other studies. A case control study initiated by Vlak et al. (2013) found that UIA patients with significantly more cardiovascular complications than RIA patients. This paradox may be related to the medication and living habits of patients with diabetes and previous cerebrovascular history. The largest case control study to date, which involved 4,701 patients with 6,411 IAs, showed that the use of a lipid-lowering agent was significantly inversely associated with RIA (Can et al., 2018). In addition, the subjects enrolled in the International Study of Unruptured Intracranial Aneurysms were compared regarding the frequency of aspirin use, and the results revealed that the patients taking aspirin regularly experienced significantly lower odds of RIA than those who never took aspirin (Hasan et al., 2011). It is interesting to note that metformin could also inhibit intracranial aneurysm formation and progression by regulating vascular smooth muscle cell phenotype switching *via* the AMPK/ACC pathway (Li et al., 2020).

At present, the models for predicting the risk of intracranial aneurysm rupture include PHASES score and UCAS score, which are still used today due to their excellent prediction ability. In this study, we introduced the parameter MAPN into a new prediction model. Finally, only four variables were included in the MAPN model, which was less than the number of variables included the PHASES score and UCAS score. At the same time, even if the variables were reduced, the AUC value of the MAPN model reached 0.799, which is slightly higher than both the AUC of PHASES score and UCAS score (0.763, 0.741). In addition, the MAPN model has the highest sensitivity and accuracy in the diagnosis of intracranial aneurysm rupture among the three models. In view of the serious consequences after aneurysm rupture, the high sensitivity of diagnostic parameters can bring more benefits to patients.

The present research has several limitations. First of all, in the derivation of the relationship between MAPN and static pressure in aneurysms, we assume constant (Newtonian) viscosity and regard the blood flow in the whole aneurysm as an incompressible constant flow fluid, but in fact, the blood flow in aneurysms is often turbulent and its state is more complex. Second, this study is a single center retrospective study, and a further multicenter prospective cohort will be ideal. In addition, stable IAS at the time of diagnosis cannot guarantee that there is no risk of rupture in the future. Furthermore, the rupture event itself may affect the morphology of IAS, which may bias the research results.

## Conclusion

MAPN as a new parameter can augment the clinical decision-making process for assessing the rupture risk of UIAs. The MAPN model constructed by introducing this parameter outperformed the UCAS score and PHASES score in IA stability assessment. The MAPN models demonstrated great potential in aiding the clinical decision-making process, and their future application in clinical practice may provide individualized and optimal management for patients with IAs.

## Data availability statement

The raw data supporting the conclusions of this article will be made available by the authors, without undue reservation.

## Ethics statement

The studies involving human participants were reviewed and approved by Ethics Committee of First Affiliated Hospital of Zhejiang University. Written informed consent for participation was not required for this study in accordance with the national legislation and the institutional requirements. Written informed consent was obtained from the individual(s) for the publication of any potentially identifiable images or data included in this article.

## Author contributions

JS, JP, and RZ contributed to conception and design of the study. JP and RZ supervision the study. JS, KH, YZ, and FW organized the database. JS, KH, and YZ performed the statistical analysis. JS and KH wrote the first draft of the manuscript. YZ, YW, and FX wrote sections of the manuscript. RM review the grammar of the article. All authors contributed to manuscript revision, read, and approved the submitted version.
