# Supplementary figures and images for: Mean arterial pressure-aneurysm neck ratio predicts the rupture risk of intracranial aneurysm by reflecting pressure at the dome
Source: Front Aging Neurosci. 2023 Feb 1;15:1082800. doi: 10.3389/fnagi.2023.1082800 (PMC9928879; doi:10.3389/fnagi.2023.1082800)

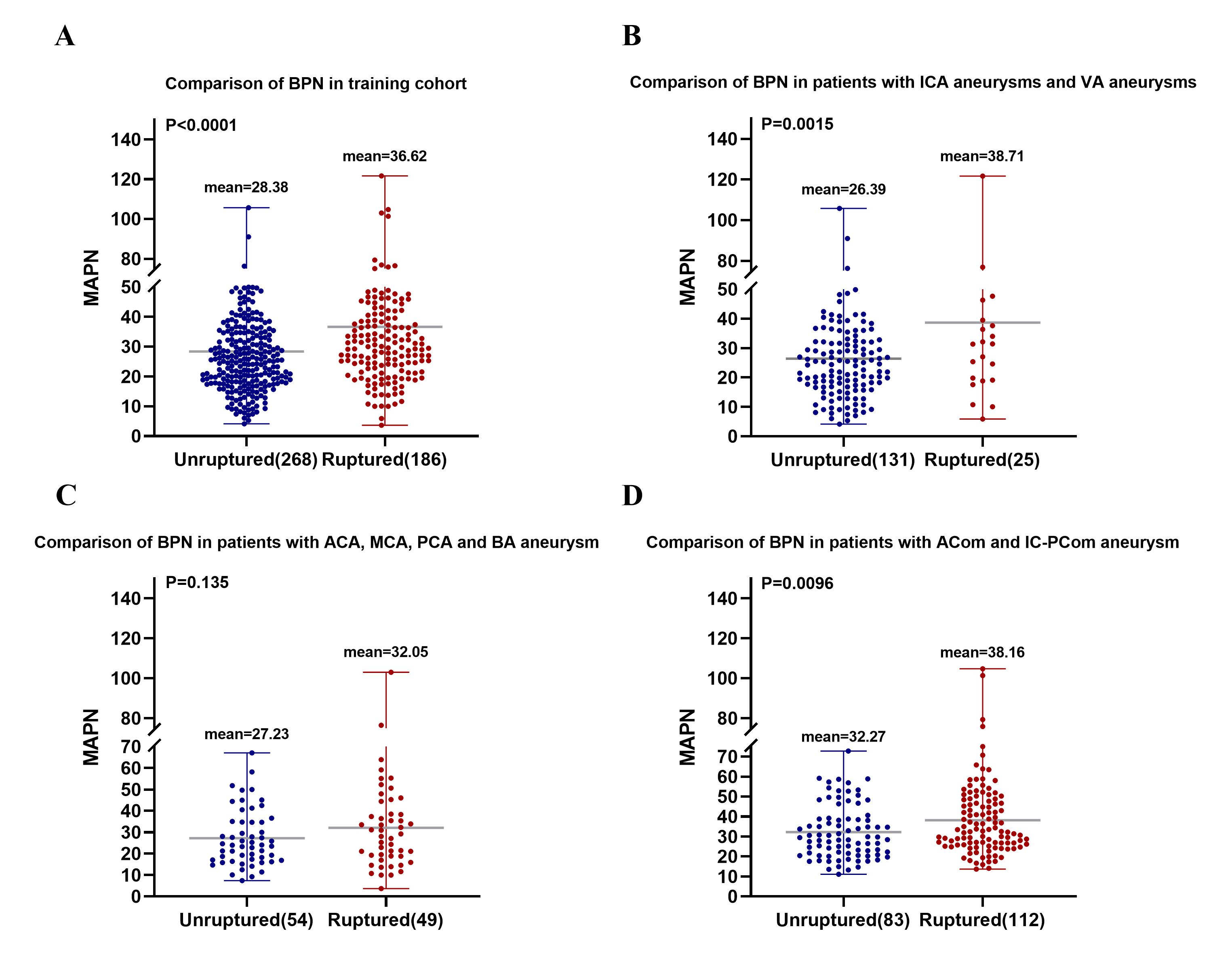

Supplement: Supplementary file 1 [file Image_1.tif]
