# Supplementary material for: Mean arterial pressure-aneurysm neck ratio predicts the rupture risk of intracranial aneurysm by reflecting pressure at the dome
Source: Front Aging Neurosci. 2023 Feb 1;15:1082800. doi: 10.3389/fnagi.2023.1082800 (PMC9928879; doi:10.3389/fnagi.2023.1082800)
Supplement: Supplementary file 2 [file Data_Sheet_1.DOCX]

**The relationship between MAPN and the static pressure of aneurysm dome**

The derivation process of the relationship between MAPN and the static pressure of aneurysm dome is shown in Figure 2. According to the principle of fluid mechanics, when fluid flows, the relationship between flow rate, pressure difference and resistance is similar to Ohm's law in electricity, where the blood flow, Q is proportional to pressure difference at both ends of blood vessel and inversely proportional to blood flow peripheral resistance Rv,^1^ which can be written as follows:

Q = △P(P1-P2)/Rv (1)

In the systemic circulatory system, Q corresponds to cardiac output (CO), Rv to total peripheral resistance, and △P to the difference between mean aortic pressure and right atrial pressure. Since the right atrial pressure is close to zero, △P is close to the mean aortic pressure (MAP). Therefore, equation (1) can be simplified as follows:

CO = MAP/Rv (2)

In this study, we refer to cardiac output as blood flow to the brain vessels. According to the flow rate equation: Q = S*V (where Q is the liquid flow, S is the pipe diameter area and V is the liquid flow rate), the intravascular flow rate can be simplified as follows:

V = MAP/(Rv*S) = MAP/(Rv*π*r^2^) (3)

According to Bernoulli's law of conservation of fluid energy，for the incompressible homogeneous fluid in the gravitational field, the equation as follows:^2^

P_1_ + 1/2(ρV_1_^2^) +ρgh_1_ = P_2_ + 1/2(ρV_2_^2^) +ρgh_2_  (4)

where p, ρ, v are the static pressure, density and velocity of the fluid respectively. h is vertical height; g is the acceleration of gravity. Previous hemodynamic researches have confirmed that the blood flow velocity at the aneurysm dome is extremely slow,^3-5^ which can almost be regarded as a stagnant state, therefore, the equation is simplified as follows:

P_1_ + 1/2(ρV_1_^2^) + ρgh_1_ = P_1_ + 1/2ρ$*(\frac{\mathrm{MAP}}{Rv * \pi*r^{2}})$^2^ + ρgh_1_ ≈ P_2_ + ρgh_2_ (5)

P_2_ **∝** $\frac{MAP}{r}$

1. Kubicek. WG, From. AH, Patterson. RP, Witsoe. DA, Castaneda. A, Lillehei. RC, et al. Impedance cardiography as a noninvasive means to monitor cardiac function. *J Assoc Adv Med Instrum*. 1970;4:79-84

2. POPP R, TEPLITSKY I. Lessons from in vitro models of small, irregular, multiple and tunnel-like stenoses relevant to clinical stenoses of valves and small vessels. 1989;13:716-722

3. Czaja B, Zavodszky G, Azizi Tarksalooyeh V, Hoekstra AG. Cell-resolved blood flow simulations of saccular aneurysms: Effects of pulsatility and aspect ratio. *J R Soc Interface*. 2018;15

4. Wang. Q, Wang. W-z, Fei. Z-m, Liu. Y-z, Cao. Z-m. Simulation of blood flow in intracranial ica-pcoma aneurysm via computational fluid dymamics modeling. *Journal of Hydrodynamics*. 2009;21:583–590

5. Steinman DA, Pereira VM. How patient specific are patient-specific computational models of cerebral aneurysms? An overview of sources of error and variability. *Neurosurg Focus*. 2019;47:E14
